# Peripheral Nerve Stimulation Optimized Pulses for EPI (POPE) allows high-resolution fMRI

**DOI:** 10.64898/2026.07.22.739360

**Published:** 2026-07-27

**Authors:** Renzo (Laurentius) Huber, Dominik Rattenbacher, Bastien Guerin, Haotian Hong, Alessandra Pizzuti, Omer Faruk Gulban, Tina (Wei-Ching) Lo, Azma Mareyam, Kyle Droppa, Jinting Yao, Cole Analoro, Paul Wighton, David Feinberg, Lawrence L. Wald, Rüdiger Stirnberg

## Abstract

**Purpose:**

Recent improvements in MRI gradient design and amplifiers, advanced MRI scanners are now routinely utilizing slew rates of several hundred T/m/s. However, full gradient performance cannot be exploited in high-resolution EPI due to peripheral nerve stimulation (PNS) limits. We aim to characterize and mitigate these PNS constraints using a simple sequence modification: PNS-optimized EPI gradient pulse shapes.

**Methods:**

PNS-Optimized Pulses for EPI (POPE): we selectively reduce the slew rate of gradient pulses at periods of high predicted PNS spikes, while leaving the rest of the waveform unchanged. PNS sensation was evaluated.

**Results:**

POPE allows 7%-35% faster imaging of EPI protocols resolutions of 1mm-0.3mm resolutions without exceeding predicted PNS. With such improvements, POPE allows robust 0.3 mm isotropic fMRI protocols that would have exceeded safety limits without it.

**Conclusion:**

POPE facilitates locally precise fMRI activation mapping on clinical 7T scanners at spatial resolution that were previously unattainable due to PNS limitations.

## Full Text Availability

The license terms selected by the author(s) for this preprint version do not permit archiving in PMC. The full text is available from the preprint server.

